# ﻿Resurrection of *Leucobryumscalare* Müll.Hal. ex M.Fleisch. (Bryophyta, Leucobryaceae) based on phylogenetic and morphometric evidence

**DOI:** 10.3897/phytokeys.222.98990

**Published:** 2023-03-20

**Authors:** Patsakorn Tiwutanon, Kasidis Chaiyasut, H. Thorsten Lumbsch, Ekaphan Kraichak

**Affiliations:** 1 Department of Botany, Faculty of Science, Kasetsart University, 50 Ngamwongwan Road, Chatuchak, Bangkok, 10900, Thailand; 2 Science Division, Mahidol University International College, Mahidol University, 999 Salaya, Phutthamonthon, Nakhon Pathom 73170, Thailand; 3 Science & Education, The Field Museum, 1400 South Lake Shore Drive, Chicago, Illinois, USA; 4 Biodiversity Center, Kasetsart University, 50 Ngamwongwan Road, Chatuchak, Bangkok, 10900, Thailand

**Keywords:** Bryophytes, classification, mosses, revision, tropical biodiversity

## Abstract

*Leucobryumscalare* was described in 1904 but its taxonomic status has been disputed, being reduced to a variety of *Leucobryumaduncum* or synonymized with *Leucobryumaduncum*. The taxonomic confusion of this taxon has remained unresolved. Hence, we revisited the taxonomic status of the taxon using phylogenetic and morphometric approaches. A total of 27 samples from Leucobryumaduncumvar.aduncum and Leucobryumaduncumvar.scalare were used to generate data from four markers, including ITS1, ITS2, *atpB*-*rbcL* spacer, and *trnL*-*trnF*. The concatenated dataset was used to reconstruct a phylogenetic tree. Both qualitative and quantitative morphological characters were measured and analyzed with Principal Component Analysis (PCA) and PERMANOVA. The results showed that the two taxa are closely related but they are reciprocally monophyletic. Both qualitative and quantitative characters could also separate Leucobryumaduncumvar.scalare from Leucobryumaduncumvar.aduncum as shown with PCA and PERMANOVA. We propose the resurrection of the species rank for *Leucobryumscalare* as separate from *Leucobryumaduncum*. This work highlights the need for a more thorough revision of *Leucobryum* to clarify the actual level of diversity in this genus.

## ﻿Introduction

Bryophytes are small land plants with simple morphology and tend to be widely distributed (Schofield and Crum 1972). Many recent phylogenetic studies have demonstrated that taxa with overlapping distributions and indistinct morphologies often consist of two or more cryptic taxa (Shaw et al. 1988; Miwa et al. 2009; Hedenäs 2020). Cryptic species in bryophytes have been attributed to the recent divergences, stasis, parallelisms, reductions, and convergences in morphological characters (Vanderpoorten and Shaw 2010; Renner 2020). With increased access to molecular data, more cases of cryptic species have been identified and have led to the description of new or resurrection of previously disregarded taxa (Shaw 2001; Renner 2020). Recognizing cryptic species is essential for understanding species diversity and speciation rates, which are essential for understanding evolutionary processes and developing effective conservation strategies (Struck et al. 2018).

Similar to many moss genera, the moss genus *Leucobryum* Hampe has been shown to include several cryptic species (Oguri et al. 2006; Oguri et al. 2010; Oguri et al. 2013; Bonfim Santos and Stech 2017). The genus currently includes about 80–100 species worldwide with predominantly temperate and tropical distribution (Eddy 1990; Enroth 1990; Klazenga 2012). The important characters are white to whitish green in color and forming cushion-like colonies. Leaves are packed in a spiral arrangement and are composed of one median row of chlorophyllose cells alternated with two-row hyalocytes in the cross-section. Sporophytes have an inclined cylindrical capsule and long-rostrate operculum. (Gradstein et al. 2001; Yamaguchi 1993). Although several taxonomic studies on *Leucobryum* are available for several countries in Southeast Asia (Gangulee 1971; Eddy 1990; Enroth 1990; Yamaguchi 1993; Lin and He 1999), species classification and identification of the species remain difficult. Many species in this genus exhibit a high degree of morphological variations and overlaps among species, causing taxonomic confusion (Enroth 1990).

**Figure 1.**
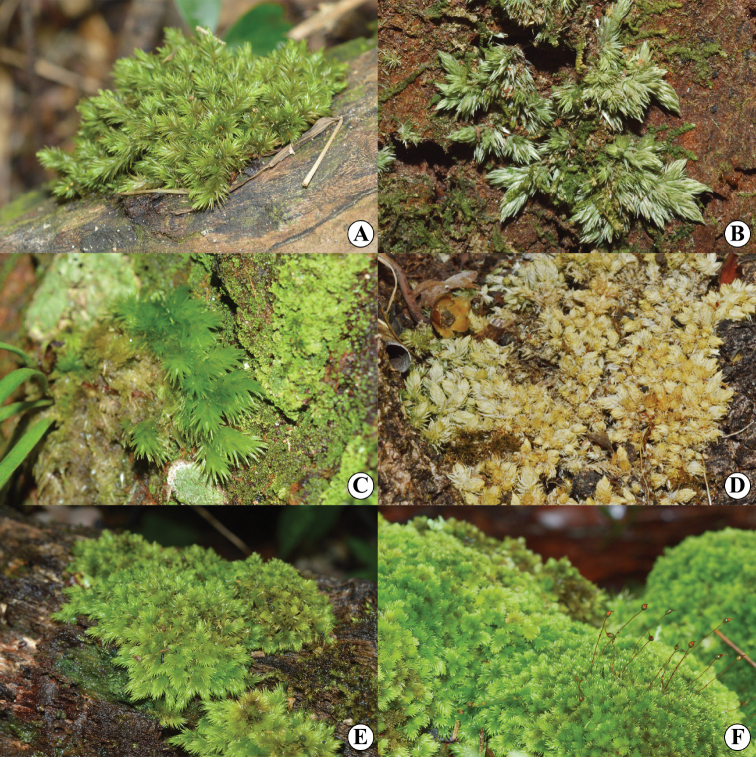
Variation of different population in *Leucobryumaduncum* and *L.scalare***A, C, E***Leucobryumaduncum***B, D, F***Leucobryumscalare*.

Among *Leucobryum* species in Southeast Asia, *Leucobryumaduncum* Dozy & Molk. and *L.scalare* Müll.Hal. ex M.Fleisch. are the most problematic due to their broadly overlapping morphologies and distributions (Fig. 1). *Leucobryumaduncum* was first described in 1854 based on a type specimen collected in Java, Indonesia (Dozy and Molkenboer 1854). The name *L.scalare* appeared in 1900 in Édouard Gabriel Paris’s Index Bryologicus, ascribed to Karl Müller, who cited specimens from the Philippines. Max Fleischer later officially described the name in 1904 (Paris 1900; Fleischer 1904). In 1990, Alan Eddy examined specimens from Malaysia and reduced the name to the variety rank as L.aduncumvar.scalare (Müll.Hal. ex. Fleisch.) A. Eddy (Eddy 1990). In the same year, however, the name *L.scalare* was also synonymized with *L.aduncum* by Johannes Enroth, who studied Leucobryaceae in the Huon Peninsula, Papua New Guinea. He noticed that the relative length of the inner perichaetial leaves around the sporophytes of *L.scalare* was around the same size as that of *L.aduncum* (Enroth 1990). Both classifications (as variety or as synonym) have been used since then interchangeably. No detailed morphological study or molecular work has been conducted to clarify the position of the name *L.scalare*.

Therefore, we aim to clarify the taxonomic status of Leucobryumaduncumvar.scalare based on morphology and molecular phylogeny. We obtained detailed morphological data from the herbarium collection and new samples of L.aduncumvar.aduncum and L.aduncumvar.scalare from Southeast Asia to perform statistical classification. We also generated DNA sequences from these specimens to reconstruct a molecular phylogeny to better understand the taxonomic status of *L.scalare*.

## ﻿Materials and methods

### ﻿Plant samples

A total of 27 samples of L.aduncumvar.aduncum (13 samples) and L.aduncumvar.scalare (14 samples), collected from various locations from 2015–2020 (Appendix 1), were used for reconstructing phylogeny and morphometrics. The specimens were identified based on their morphology as described in relevant taxonomic literature (Eddy 1990; Yamaguchi 1993; Lin and He 1999).

### ﻿Morphology and morphometrics

**Figure 2.**
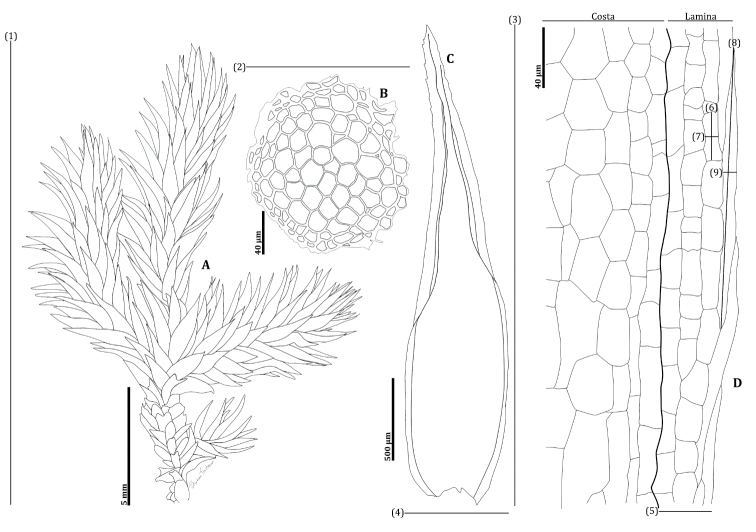
Diagram showing the morphological characteristics **A** gametophyte **B** cross-section of stem **C** leaf **D** costa and lamina. Measuring the quantitative characteristics **(1)** gametophyte height (**2)** stem diameter **(3)** leaf length **(4)** leaf width **(5)** lamina width **(6)** lamina cell length **(7)** Lamina cell width **(8)**border cell length **(9)** border cell width.

Gametophytes were investigated for qualitative and quantitative characters using a Motic SMZ-171 stereomicroscope and Motic BA310E biological microscope. The terminology of morphological characters primarily followed those from Yamaguchi (1993) and Malcolm and Malcolm (2006). The quantitative characters (Fig. 2) included gametophyte height (cm), stem diameter (µm), leaf length (mm), leaf width (mm), leaf ratio (length to width ratio), lamina width (µm), lamina cell length (µm), lamina cell width (µm), border cell length (µm) and border cell width (µm). A Canon EOS500D digital camera and the EOS Utility V. 3 software for the automatic image were used to take images of leaves and cells. Measurements were taken from these leaf and cell images using the Fiji V. 1.53s software (Schindelin et al. 2012).

Each quantitative character of the two taxa was compared visually with a boxplot and statistically using a Wilcoxon’s test (David 1972). Then, all quantitative data were subjected to a Principal Component Analysis (PCA) to determine whether the combined information corresponded with the two taxa. PERMANOVA was used to test for differences between the two taxa with a multivariate dataset. All morphometric analyses were performed in the R program V. 3.6.1 (R Core Team 2019).

### ﻿Phylogenetic analyses

Genomic DNA was extracted from the samples using the NucleoSpin Plant II Kit (Macherey-Nagel GmbH & Co. KG, Germany) following the manufacturer’s user manual. A sample was homogenized by grinding dried samples in liquid nitrogen. Four regions, including ITS1, ITS2, *atpB*-*rbcL* spacer, and *trnL*-*trnF*, were amplified with Polymerase Chain Reactions (PCR) using the primers and conditions in Bonfim Santos and Stech (2017) (Appendix 2).The cleaned PCR products were sent to Macrogen Inc. (www.macrogen.com, Seoul, South Korea) to perform Sanger sequencing. The chromatograms and nucleotide sequence data were then sent back for manual assembly using the Geneious Prime v.2022.0.1 (www.geneious.com).

The corresponding sequences of *L.candidum* (Brid. ex P. Beauv.) Wilson (*HIRO 203728*: AB285170, AB288196, AB742389) and *L.chlorophyllosum* Müll. Hal. (*HIRO 140710*: AB125291, AB124792, AB742390; *HIRO 140820*: AB763361, AB739636, AB742391; *MAK B119208*: AB763362, AB739637, AB742392), available in the NCBI database, were selected as an outgroup based on Bonfim Santos and Stech (2017). The sequences of *L.aduncum* in the NCBI database, the newly generated *L.aduncum* sequences of two varieties and the outgroup sequences (Appendix 1) were aligned to their corresponding homologous position using the MUSCLE algorithm (Edgar 2004) available in Geneious Prime v.2022.0.1 (Biomatters Ltd., Auckland, New Zealand) (https://www.geneious.com). Phylogenetic trees were constructed using the maximum likelihood (ML) and Bayesian inference (BI) methods available on HPC, Faculty of Science, Kasetsart University, Thailand. ML trees were constructed using RAxML v. 8.2.12 (Stamatakis 2014), and the branch support value of the ML tree was estimated by the bootstrap algorithm with 1,000 bootstrap replicates. BI tree was constructed by MrBayes v.3.2 (Ronquist et al. 2012) with the Bayesian posterior probabilities calculated using the Metropolis-coupled Markov Chain Monte Carlo (MCMCMC) method. Four chains (three heated and one cold) with the temperature set to 0.2 were run for 20,000,000 generations, with chains sampled every 1000 trees. Twenty-five percent of the posterior trees were discarded as burn-in. The phylogenetic trees were then visualized, adjusted, and produced using Figtree ver. 1.4.4 (Rambaut 2018). Bootstrap support (BS) of 70 or greater from the ML analysis and posterior probability (PP) of 0.9 or greater from the BI analysis were considered strong support for a clade.

## ﻿Results

### ﻿Morphology and morphometrics

Variation of Qualitative Characters – Gametophytes of L.aduncumvar.scalare are relatively small and often form a compact cushion with dense branches. The habitat is in open sites on tree trunks or logs, rarely on branches or rocks. Meanwhile, the tuft form of L.aduncumvar.aduncum is small- to medium-sized with little branching, but usually, several branches can be found in small gametophytes. The habitat is in shaded sites on logs, tree trunks, humus, or rocks. Both taxa lack a central strand. When dry, the plants are yellowish green to whitish green and brown (Figs 6–9). Leucobryumaduncumvar.aduncum and L.aduncumvar.scalare are readily distinguished by their leaf arrangement, orientation, and shape. The leaves of L.aduncumvar.scalare are spirally arranged and closely imbricate, forming a conical point at the shoot apex, especially when dry. The leaves are erect but sometime falcate-secund when growing near the substrate. The leaf shape is lanceolate to narrowly lanceolate with an oblong to ovate base (Figs 8, 9). In contrast, the leaves of L.aduncumvar.aduncum are not spirally arranged and do not form a conical point. The leaves are markedly falcate-secund and sometimes slightly erect. The leaf shape is lanceolate with an ovate to oblong base (Figs 6, 7).

**Figure 3.**
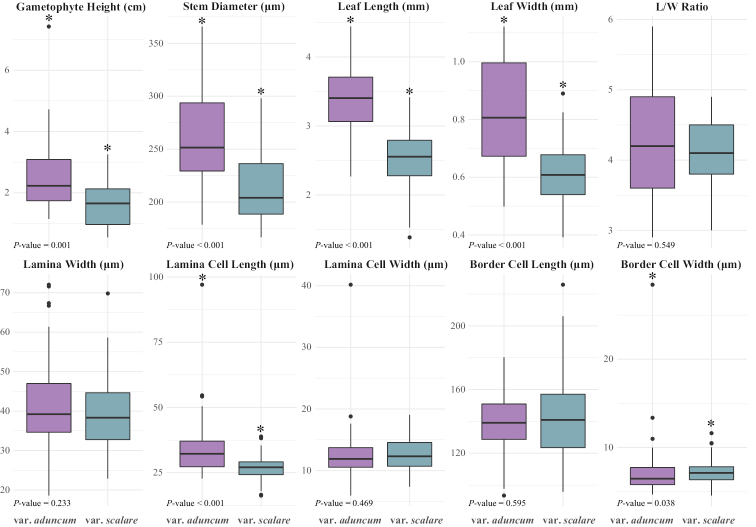
Boxplot showing variation on the ten quantitative characters in the two varieties of *Leucobryumaduncum*. The boxes represent the data from the 25^th^ to 75^th^ percentiles, with the median at the line in the box. The black dots outside the bar represent the “outlier” data points. All *P*-values are from Wilcoxon’s test between the two varieties, and the two varieties are considered significantly different in that trait at *P*-value ≤ 0.05.

Variation of Quantitative Characters – Leucobryumaduncumvar.scalare and L.aduncumvar.aduncum were significantly different in the following six morphological characters: gametophyte height, stem diameter, leaf length, leaf width, lamina cell length, and lamina cell width (Fig. 3). Gametophyte height, stem diameter, leaf length, leaf width, and lamina cell length of L.aduncumvar.scalare were smaller than those of L.aduncumvar.aduncum. The other four quantitative characters (leaf ratio, lamina width, lamina cell width, and border cell length) showed no significant differences between the two taxa.

**Figure 4.**
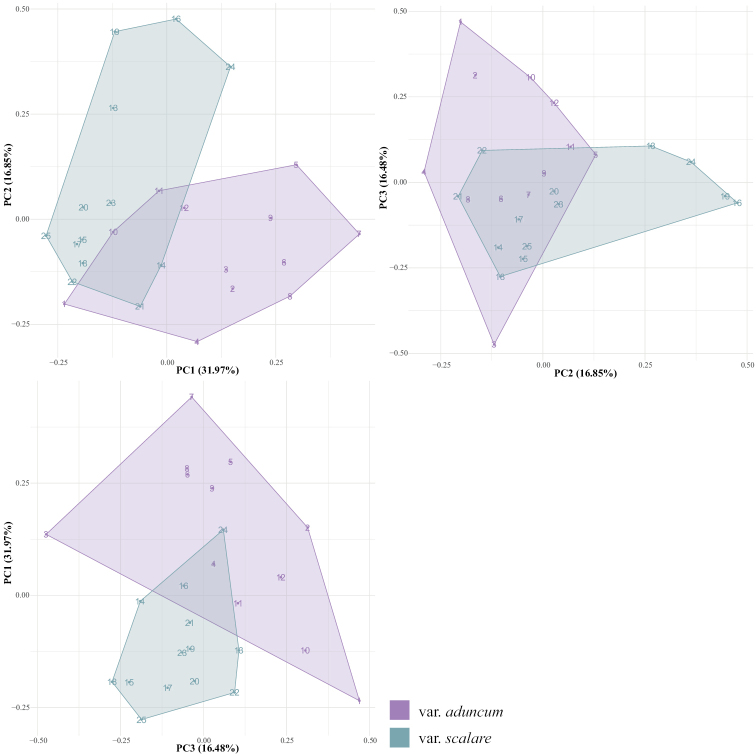
Principal component analysis (PCA) plot based on the ten quantitative characters of the two varieties of *Leucobryumaduncum*. The groups are significantly different at *P*-values = 0.001 (PERMANOVA).

PCA Analysis – The first three principal components (PC I, II, III) from the analysis with ten morphological characters accounted for 31.97%, 16.85%, and 16.48% of the variance, respectively (Fig. 4). All combinations of the first three principal components showed that L.aduncumvar.scalare were separated from L.aduncumvar.aduncum. The PERMANOVA test showed that these two taxa were significantly different from each other in their morphologies (*F* = 5.53, *P*-values = 0.001).

### ﻿Phylogenetic analyses

**Figure 5.**
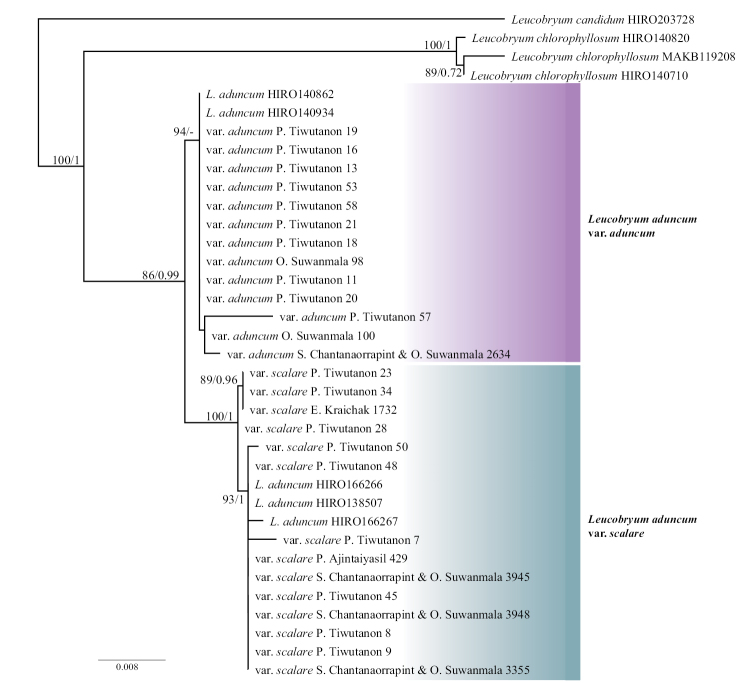
Maximum likelihood consensus tree of 32 representatives of the two varieties of *Leucobryumaduncum* based on nuclear and chloroplast DNA sequences (ITS1, ITS2, *atpB-rbcL* spacer, and *trnL-trnF*). Branch support values are from Bayesian inference (BI) and Maximum likelihood (ML) analyses of the same alignment. The Bootstrap (BS; ≥ 70%) values and Posterior probabilities (PP; ≥ 0.95) are shown at the nodes, respectively, with non-matching clades using different analyses indicated by ‘–’. The tree was outgroup-rooted by *L.candidum* and *L.chlorophyllosum*.

A total of 106 new sequences from nuclear and chloroplast markers (ITS1, ITS2, *atpB*-*rbcL* spacer, and *trnL*-*trnF* regions) were generated in the current study and aligned with existing sequences of *L.aduncum* sequences (five samples) and the outgroup (*Leucobryumcandidum* and *L.chlorophyllosum*) available in the NCBI database (Appendix 1). A matrix of 1,644 nucleotide characters was aligned, and 1,310 characters (79.7%) in the alignment were conserved sites. For the nuclear regions, the aligned sequences of ITS1 and ITS2 had a length of 376 base pairs with 219 constant characters (58.2%) and 283 base pairs with 232 constant characters (82%), respectively. For the chloroplast regions, the aligned sequences of *atpB*-*rbcL* spacer and *trnL*-*trnF* had a length of 579 base pairs with 563 constant characters (97.2%) and 406 base pairs with 376 constant characters (92.6%), respectively. Because the topologies of the phylogenetic trees constructed from the nuclear and chloroplast regions did not show any strongly supported conflicts in both the ML and BI analyses, only the topology of the ML consensus tree was shown here with the posterior probability of the BI analysis added (Fig. 5). The samples of *L.aduncum* were split into two well-supported sister clades: Leucobryumaduncumvar.aduncum clade and L.aduncumvar.scalare clade (BS 86%, PP 0.99). This result demonstrated that L.aduncumvar.scalare and L.aduncumvar.aduncum were reciprocally monophyletic and could be considered two separate species. Given the support from morphological and molecular data, we propose the resurrection of the name *Leucobryumscalare* Müll.Hal. ex M.Fleisch. at the species level with the revised descriptions as follows.

## ﻿Taxonomic treatment

### ﻿ Leucobryum aduncum

Taxon classificationPlantaeDicranalesLeucobryaceae

Dozy & Molk., Pl. Jungh. 3: 319. 1854.

BEFF4DB6-0C52-54A8-8198-2D67748B3B7A

Figs 6 , 7

#### Type.

Indonesia. Java: *Junghuhn s.n.* (lectotype, designated by Yamaguchi 1993, pg. 31: L; isolectotype: L).

#### Description.

Gametophytes usually form tufts, small to medium size, 1–8 cm long with leaves, yellowish green to whitish green, brown when dry. Stems erect, less branched, usually with several branches in small gametophytes; central strand absent in cross-section of stems. Leaves falcate-secund, sometimes slightly erect, 2.3–4.4 mm long, 0.5–1.1 mm wide, lanceolate, gradually narrowed to subtubulous point from ovate to oblong base, cuneate, margin entire, acute at the apex, undulate and spinosely prorate on abaxial surface; laminae consisting 1–4 rows, lamina cells quadrate to narrowly rectangular, thin-walled; borders consisting 1–3 rows, border cells linear to fusiform, thin-walled; in cross-section of leaf base hyalocytes in 1–2 rows on the adaxial side and 2–3 rows on abaxial side, if 3 rows, usually consisting 1 large row and 2 small rows; adaxial and abaxial side of median leaves consisting 1 row. Dioicous. Perichaetia terminal on short branches; perichaetial leaves around sporophytes shorter than ordinary leaves, ovate to lanceolate, abruptly narrowed to the point, cucullate, acuminate at apex. Sporophytes dicranoid. Setae elongate, erect, 1.7–2.1 cm long. Capsules ovoid to ellipsoid, inclined, 1–2 mm long, 0.5–0.6 mm diameter; opercula long rostrate; peristomes dicranoid. Calyptra cucullate.

**Figure 6.**
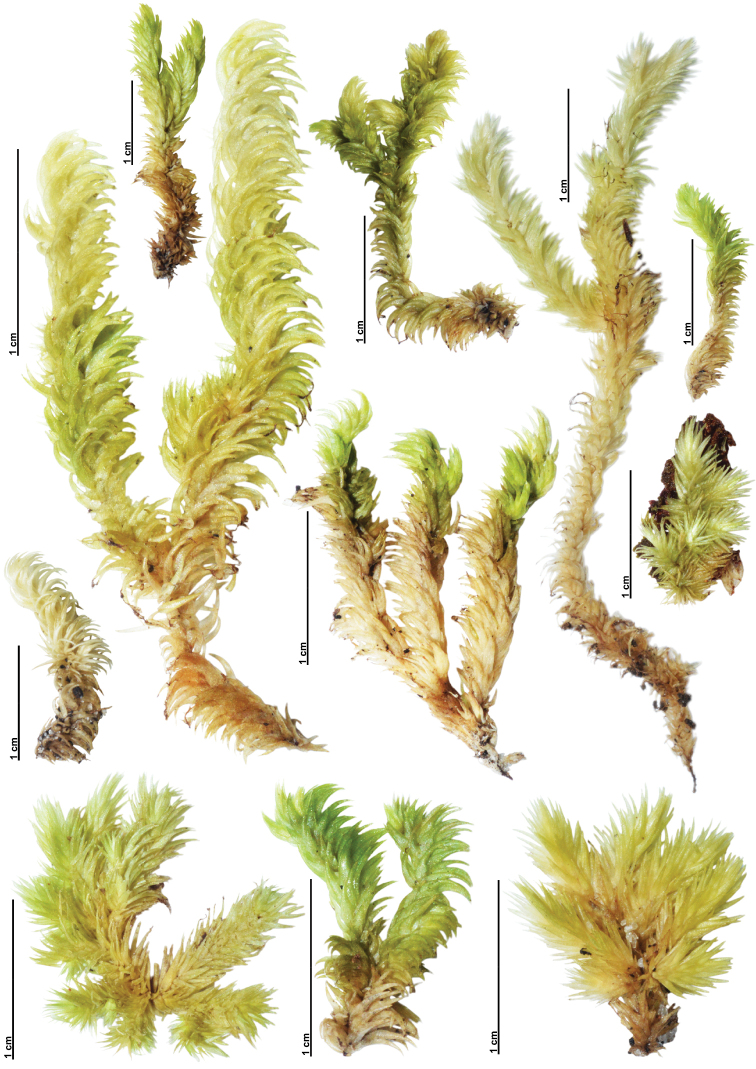
Gametophyte variation of different populations of *Leucobryumaduncum* Dozy & Molk.

**Figure 7.**
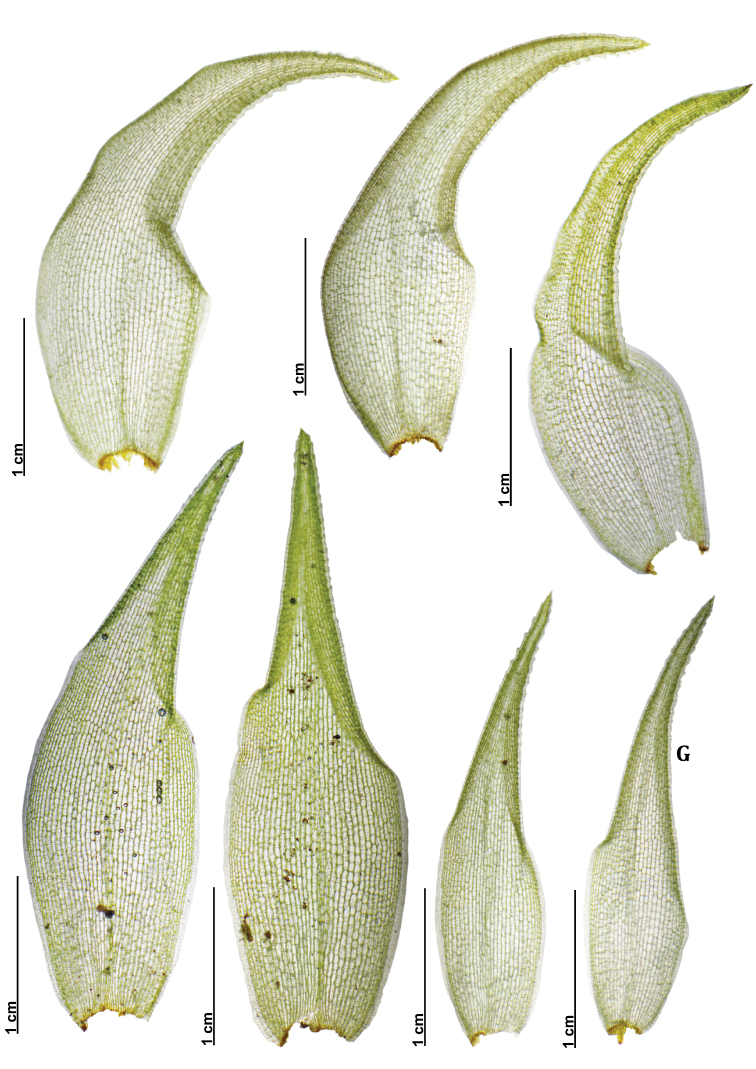
Leaf shape and size variation from different populations of *Leucobryumaduncum* Dozy & Molk.

#### Habitat.

Usually found in more shaded sites with high moisture, on logs, tree trunks, humus, and rocks.

#### Distribution.

Mainland China, India, Nepal, Sri Lanka, Thailand, Laos, Cambodia, Vietnam, Peninsular Malaysia, Singapore, Philippines, Borneo, Sulawesi, Sumatra, Java, Lesser Sunda Islands, Seram, New Guinea (Yamaguchi 1993; He 1995).

#### Illustrations.

Eddy 1990 (fig. 169A–F); Yamaguchi 1993 (Pls. XV, 1–16; XVI, 1–11).

### ﻿ Leucobryum scalare

Taxon classificationPlantaeDicranalesLeucobryaceae

Müll.Hal. ex M.Fleisch., Musci Buitenzorg 1: 143. 1904.

3E5703D5-1DA3-5AAA-A493-0209F3590886

Figs 8 , 9

Leucobryum aduncum var. scalare (Müll.Hal. ex M.Fleisch.) A.Eddy, Handb. Males. Mosses 2: 11. 1990, syn. nov.

#### Type.

The Philippines. Luzon, Benguet: 5000 ft. alt., *W. Micholitz 173* (lectotype, designated by Yamaguchi 1993, pg. 33: FH! [00290301]).

*Leucobryumperichaetiale* Dixon, J. Siam Soc., Nat. Hist. Suppl. 9(1): 11. 1932. Type: Thailand. Northern, Doi Suthep, ca. 1500 m alt., 6 Sept. 1914. *Kerr s.n.* (*in herb. Dixon*, *ref. no. 8*) (holotype: BM [BM000866895]).

*Leucobryummicroleucophanoides* Dixon ex A. Johnson, Gard. Bull. Singapore 20: 333. f. 9: m, 12. 1964. Type: Peninsular Malaysia. Kedah, Inchang Estate, on the decaying trunk, 24 Apr. 1940. *Spare s.n.* (*in herb. Dixon*, *ref. no. 2941*) (holotype: BM [BM000866907]).

#### Description.

Gametophytes usually form a small compact cushion, 0.5–3.3 cm long with leaves, yellowish green to whitish green, and brown to dark brown when dry. Stems erect, with many short branches, usually very dense; central strand absent in cross-section of stems. Leaves spiral and closely imbricate, forming a conical point at shoot apex when dry, 1.4–3.4 mm long, 0.4–0.9 mm wide, lanceolate to narrowly lanceolate, gradually or abruptly narrowed to subtubulous point from oblong to ovate base, cuneate, margin entire, acute at the apex, undulate and spinosely prorate on abaxial surface, sometimes undulate and papillosely prorate; laminae consisting 1–3 rows, lamina cells quadrate to rectangular, thin-walled; borders consisting 1–3 rows, border cells linear to narrowly fusiform, thin-walled; in cross-section of leaf base hyalocytes in 1–2 rows on the adaxial side and 2–3 rows on abaxial side, if 3 rows, usually 2 large rows and 1 small row; adaxial and abaxial side of median leaves consisting 1 row. Dioicous. Perichaetia terminal on short or long lateral branches; perichaetial leaves around sporophytes longer than ordinary leaves, ovate to lanceolate, abruptly slender to the point, cucullate, acuminate at apex. Sporophytes dicranoid. Setae elongate, erect, 1.5–1.7 cm long. Capsules subglobose to ovoid, inclined, 1.0–1.5 mm long, 0.4–0.5 mm diameter; opercula long rostrate; peristomes dicranoid. Calyptra cucullate.

**Figure 8.**
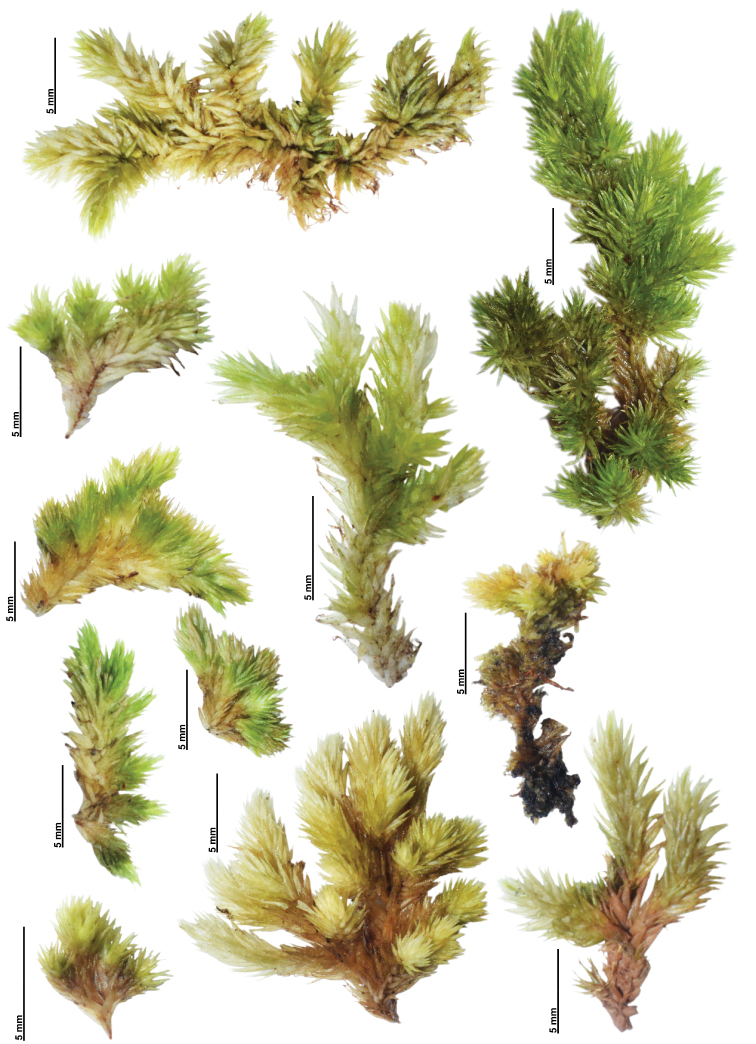
Gametophyte variation of different populations of *Leucobryumscalare* Müll.Hal. ex M.Fleisch.

**Figure 9.**
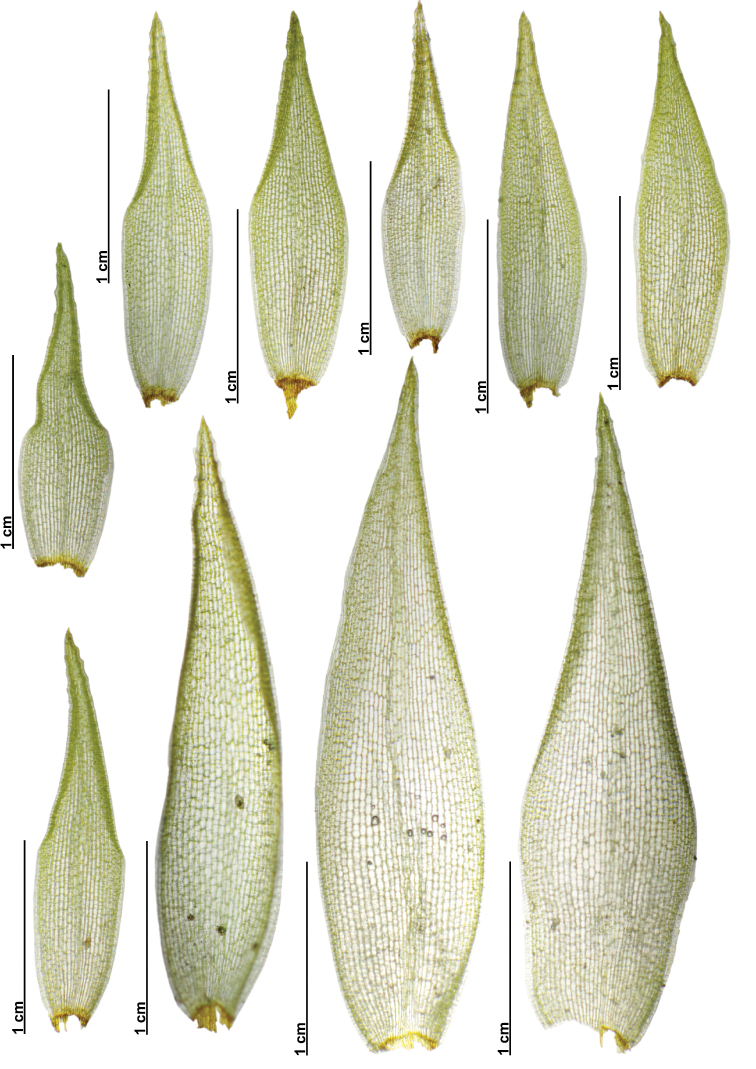
Leaf shape and size variation of different populations of *Leucobryumscalare* Müll.Hal. ex M. leisch

#### Habitat.

Usually found in more open sites, on logs, tree trunks, tree bases, branches, and rocks.

#### Distribution.

Mainland China, India, Sri Lanka, Myanmar, Thailand, Laos, Cambodia, Vietnam, Peninsular Malaysia, Philippines, Borneo, Sumatra, Java, Seram, New Guinea, and New Caledonia (Yamaguchi 1992; He 1995).

#### Illustrations.

Eddy 1990 (fig. 170A–E); Yamaguchi 1993 (Pls. XIX, 1–28; XX, 1–11; XXI, 1–15; XXII, 1–26).

##### ﻿Key to the species

**Table:** 

1	Gametophytes small to medium-sized, usually forming tufts, less branched, usually several branching in small gametophytes. Leaves falcate-secund, sometimes slightly erect, not forming a conical point at the shoot apex when dry	***Leucobryumaduncum***
–	Gametophytes small sized, usually forming a compact cushion, with many short branches, usually very dense. Leaves erect, sometimes falcate-secund when close to the substrate, arranged in spiral and closely imbricate, forming a conical point at shoot apex when dry	***Leucobryumscalare***

## ﻿Discussion

*Leucobryumaduncum* and *L.scalare* have long been problematic taxa due to their overlapping geographical distributions and indistinct morphological characters (Fig. 1). Chopra (1975) noticed that these taxa were very similar and needed more detailed study. In 1990, Alan Eddy regarded the specimens of *L.scalare* as the ‘scalare’ phenotype from the environment with long exposures to light and periodic desiccation. Then, Eddy reduced *L.scalare* to a variety of *L.aduncum* (Eddy 1990). This treatment of *L.scalare* as a variety was later accepted by Yamaguchi (1993). He reported that the inner perichaetial leaves around sporophytes of *L.scalare* were longer than vegetative leaves, while *L.aduncum* have inner perichaetial leaves as long as, or a little shorter than, vegetative leaves. This slight difference was recognized as a difference between varieties and insufficient to separate *L.scalare* at the species level (Yamaguchi and Iwatsuki 1987; Yamaguchi 1993). In the same year that Alan Eddy reduced *L.scalare* to the variety level, Johannes Enroth also reported a study of Leucobryaceae in Indonesia and Papua New Guinea. He noticed that the relative length of the inner perichaetial leaves around the sporophytes of *L.scalare* was similar in size to those in *L.aduncum* (Enroth 1990). With this observation, Enroth treated *L.scalare* as a synonym with *L.aduncum*. Enroth’s treatment of *L.scalare* as a synonym of *L.aduncum* has been accepted by many bryologists (Gao 1994; Crosby et al. 1999; Thouvenot and Bardat 2010). Despite the general adoption of Enroth’s concept of *L.aduncum* (with *L.scalare* as a synonym), L.aduncumvar.scalare following Eddy is still widely used today, especially in floras and reports from South and Southeast Asia (Lin and He 1999; Mamalo and Supremo 2007; Biju and Daniels 2017), due to somewhat recognizable morphological characters. Still, no taxonomic revision since Yamaguchi in 1993 has attempted to clarify the position of this taxon.

We here propose reinstating the name *L.scalare* at the species level following our morphological and phylogenetic analyses. Our morphological and morphometric studies showed that *L.aduncum* was generally larger than *L.scalare* (Fig. 3). However, many quantitative characters still overlapped between the two taxa and were unsuitable as taxonomic characters. Other environmental factors and the age of plants may influence these variable characters. The PCA and PERMANOVA tests showed that these two taxa were separate in their morphological space and should be recognized as separate taxonomic units of the same rank. The recognition of *L.scalare* as a distinct taxon was consistent with the previous observation by Eddy. He noticed that the leaf characters were sufficiently different from other taxa but decided that *L.scalare* should be a variety of *L.aduncum* (Eddy 1990). As for the use of inner perichaetial leaves in Yamaguchi and Iwatsuki (1987) and Enroth (1990), the current study showed that the inner perichaetial leaves were not the most reliable character, as they were hard to find in the specimens. We could not definitively conclude whether these traits differ between the two taxa. Our limited data on perichaetial leaves were consistent with those from Yamaguchi and Iwatsuki (1987). Even though Enroth (1990) found the inner perichaetial leaves to be similar in these two taxa, this character is quite difficult to verify in most specimens. Other than the perichaetial leaves, the other gametophytic characters showed consistent differences between *L.aduncum* and *L.scalare*, allowing bryologists to make a clear, unequivocal identification of these taxa.

The previous confusion over the taxonomic status of *Leucobryumscalare* could be the result of cryptic species within the species complex. Cryptic species are taxonomic groups that are similar in morphology due to their short divergence times despite a clear genetic distinction (Struck et al. 2018; Renner 2020). Many cases of cryptic species have been reported in bryophytes, vascular plants, fungi, and lichens (Shaw 2001; Bickford et al. 2007; Crespo and Pérez-Ortega 2009; Renner 2020). The discoveries of genetically distinct groups within the morphologically similar complex have driven more detailed morphological studies to find the defining characteristics of the observed genetic groups, which subsequently enhance our ability to perform taxonomic revision (Renner 2020). Several species complexes in *Leucobryum* have been recognized (Patterson et al. 1998; Vanderpoorten et al. 2003; Oguri et al. 2006; Oguri et al. 2008; Oguri et al. 2013). For example, *L.glaucum* (Hedw.) Ångstr. and *L.albidum* (Brid. ex P.Beauv.) Lindb. from eastern North America have similar morphological characters and broadly overlapping geographical distributions (circumboreal for *L.glaucum* and amphiatlantic to North America and Europe for *L.albidum*). *L.glaucum* and *L.albidum* do not require significantly different environmental conditions. These are common species in various woodland habitats ranging from xeric, sandy sites to swamp forests. The only difference between the two species was the size. However, RFLP analyses of nuclear ribosomal DNA showed that *L.albidum* is genetically distinct from *L.glaucum* (Patterson et al. 1998). The case of *L.albidum*-*L.glaucum* complex demonstrated that morphological and ecological differences were not the sole determinants of the species boundary. Additional data from molecular markers and detailed morphological work can help identify different taxonomic units within the complex.

In this case, *Leucobryumscalare* and *L.aduncum* could be the results of a recent divergence because of their morphological overlapping (Figs 3, 4) and short genetic distance (Fig. 5). From personal observations, variations in environmental conditions might be responsible for the difference between species. *Leucobryumaduncum* is often found growing in the shade of trees and with high moisture, while *L.scalare* is found growing on the substrate with prolonged exposure to light and periodic desiccation. The difference in the ecological niche may become one of the reproductive barriers leading to the speciation of *L.scalare* and *L.aduncum*. Further studies on their ecological differences should be conducted to ascertain the mechanisms behind the speciation event.

## Supplementary Material

XML Treatment for Leucobryum aduncum

XML Treatment for Leucobryum scalare

## ﻿Appendix 1

Voucher Specimens and GenBank numbers for ITS1, ITS2, *atpB-rbcL* spacer, and *trnL-trnF* regions in this study.

***Leucobryumaduncum***, Indonesia: Borneo, HIRO 140862, (HIRO), GenBank: AB125287, AB124781, AB742374; HIRO 140934, (HIRO), GenBank: AB763349, AB739623, AB742375. Thailand: Nakhon Nayok, Khao Yai National Park. Kog Kaeo Waterfall, *Tiwutanon 57*, (BKF), GenBank: OQ556892, OQ557103, OQ581043; *Tiwutanon 58*, (BKF), GenBank: OQ556890, OQ557101, OQ576673, OQ581052; Pha Kluai Mai Waterfall, *Tiwutanon 53*, (BKF), GenBank: OQ556891, OQ557102, OQ576674, OQ581051. Northeast s.n. *Tiwutanon 16*, (BKF), GenBank: OQ556885, OQ557096, OQ576668, OQ581045; *Tiwutanon 18*, (BKF), GenBank: OQ556886, OQ557097, OQ576669, OQ581048; *Tiwutanon 19*, (BKF), GenBank: OQ556887, OQ557098, OQ576670, OQ581046; *Tiwutanon 20*, (BKF), GenBank: OQ556882, OQ557093, OQ576665, OQ581044; *Tiwutanon 21*, (BKF), GenBank: OQ556883, OQ557094, OQ576666, OQ581053; *Tiwutanon 11*, (BKF), GenBank: OQ556880, OQ557091, OQ576663, OQ581047; *Tiwutanon 13*, (BKF), GenBank: OQ556881, OQ557092, OQ576664, OQ581056. Phangnga, Si Phang-Nga National Park. Khao Dan Trail [14°22.5'N, 101°24.54'E], *Chantanaorrapint & Suwanmala 2634*, (PSU), GenBank: OQ556888, OQ557099, OQ576671, OQ581050. Phangnga, Khao Lampi-Hat Thai Mueang National Park, Thai Mueang District. [8°29.1'N, 98°13.68'E], *Suwanmala 98*, (PSU), GenBank: OQ556889, OQ557100, OQ576672, OQ581049; *Suwanmala 100*, (PSU), GenBank: OQ556884, OQ557095, OQ576667, OQ581054. ***Leucobryumcandidum***, New Zealand: HIRO 203728, (HIRO), GenBank: AB285170, AB288196, AB742389. ***Leucobryumchlorophyllosum***, Indonesia: Borneo, HIRO 140710, (HIRO), GenBank: AB125291, AB124792, AB742390; HIRO 140820, (HIRO), GenBank: AB763361, AB739636, AB742391. Philippines: MAK B119208, (MAK), GenBank: AB763362, AB739637, AB742392. ***Leucobryumscalare***, Malaysia: Malay Peninsula, HIRO 138507, (HIRO), GenBank: AB763350, AB739624, AB742376., Sri Lanka: Nuwara Eliya District, HIRO 166266, (HIRO), GenBank: AB763351, AB739625, AB742377; HIRO 166267, (HIRO), GenBank: AB763352, AB739626, AB742378. Thailand: Chiang Mai, Chiang Dao Wildlife Sanctuary, Den Ya Khad. [19°22.38'N, 98°50.04'E], *Chantanaorrapint & Suwanmala 3355*, (PSU), GenBank: OQ556894, OQ557105, OQ576676, OQ581064. Chiang Mai, Chiang Mai Royal Agricultural Research Center (Khun Wang) Botanical Garden. [18°45.18'N, 98°55.5'E], *Tiwutanon 7*, (BKF), GenBank: OQ556906, OQ557117, OQ581055. Lampang, Doi Khun Tan National Park, Doi Khun Tan. Tat Mei Khun Tan Waterfall [18° 30.84'N, 99° 17.46'E], *Tiwutanon 8*, (BKF), GenBank: OQ556895, OQ557106, OQ576677, OQ581065; *Tiwutanon 9*, (BKF), GenBank: OQ556896, OQ557107, OQ576678, OQ581066. Loei, Phu Ruea National Park. *Tiwutanon 45*, (BKF), GenBank: OQ556901, OQ557112, OQ576683, OQ581058; *Tiwutanon 48*, (BKF), GenBank: OQ556902, OQ557113, OQ576684, OQ581057; *Tiwutanon 50*, (BKF), GenBank: OQ556903, OQ557114, OQ576685, OQ581060. Loei, Phu Kradueng National Park. Nong Pakbung [16°50.64'N, 101°41.52'E], *Ajintaiyasil 429*, (BCU), GenBank: OQ556904, OQ557115, OQ576686, OQ581059. Nakhon Nayok, Khao Yai National Park, Khao Kheow. [14°22.5'N, 101°24.54'E], *Kraichak 1732*, (BKF), GenBank: OQ556899, OQ557110, OQ576681, OQ581063; *Tiwutanon 23*, (BKF), GenBank: OQ556900, OQ557111, OQ576682, OQ581061; *Tiwutanon 28*, (BKF), GenBank: OQ556898, OQ557109, OQ576680, OQ581068; Deaw Dai Cliff. [14°21.96'N, 101°24.36'E], *Tiwutanon 34*, (BKF), GenBank: OQ556897, OQ557108, OQ576679, OQ581067. Phayao, Doi-Luang National Park. The way up to Doi Luang [19°7.86'N, 99°45.42'E], *Chantanaorrapint & Suwanmala 3948*, (PSU), GenBank: OQ556893, OQ557104, OQ576675, OQ581062; *Chantanaorrapint & Suwanmala 3945*, (PSU), GenBank: OQ556905, OQ557116, OQ576687, OQ581069.

## ﻿Appendix 2

**Table A1.** Primers for the ITS1, ITS2, *atpB-rbcL* spacer, and *trnL-trnF* regions in this study.

Region	Primers	References
**ITS1**		
Bryo18SF	5’- GGT GAA GTT TTC GGA TCG CG -3’	Hartmann et al. 2006
Bryo5.8SR	5’- TGC GTT CTT CAT CGT TGC -3’	Hartmann et al. 2006
**ITS2**		
Bryo5.8SF	5’- GAC TCT CAG CAA CGG ATA -3’	Hartmann et al. 2006
Bryo26SR	5’- AGA TTT TCA AGC TGG GCT -3’	Hartmann et al. 2006
***atpB-rbcL* spacer**		
atpB-2	5’- AGC GTT GTA AAT ATT AGG CAT CTT -3’	Hsu et al. 2013
rbcL-2	5’- ATC TTT AAC ACC AGC TTT GAA TCC AAC -3’	Hsu et al. 2013
***trnL-trnF***		
C_(M)_	5’-CGA AAT CGG TAG ACG CTA CG- 3’	Taberlet et al. 1991
F_(M)_	5’-ATT TGA ACT GGT GAC ACG AG- 3’	Frey et al. 1999
